# Reproductive Outcome After GnRH Agonist Triggering With Co-Administration of 1500 IU hCG on the Day of Oocyte Retrieval in High Responders: A Long-Term Retrospective Cohort Study

**DOI:** 10.3389/fendo.2022.826411

**Published:** 2022-04-06

**Authors:** Dzhamilyat Abdulkhalikova, Eda Vrtacnik Bokal, Martin Stimpfel, Primoz Ciglar, Sara Korosec

**Affiliations:** ^1^Division of Obstetrics and Gynaecology, Department of Human Reproduction, University Medical Centre Ljubljana, Ljubljana, Slovenia; ^2^Department of Gynaecology and Obstetrics, Ptuj General Hospital, Ptuj, Slovenia

**Keywords:** GnRH (gonadotropin-releasing hormone) agonists, hCG (human chorionic gonadotrophin), Oocyte maturation, triggering, OHSS

## Abstract

While triggering oocyte maturation with GnRH agonist (GnRHa) seems to be safe and effective in terms of the risk of developing OHSS and the number of metaphase II oocytes, it nevertheless results in luteal phase deficiency. To date, strategies have been developed in order to rescue defective luteal phase of GnRHa triggered cycles. Our study aimed to assess the reproductive outcome of GnRHa triggered cycles combined with modified luteal support (1500 IU hCG at the day of oocyte retrieval) in women with high ovarian response and to compare the outcome with hCG triggered cycles in GnRH antagonist IVF-ICSI procedures. A retrospective cohort database review of the results of GnRH antagonist IVF-ICSI cycles was conducted at a tertiary-care IVF center in Ljubljana, Slovenia. A total of 6126 cycles, performed from January 1, 2014, to December 31, 2020, were included in the final analysis. Final oocyte maturation was performed with either 5000, 6500, or 10,000 IU hCG (women with normal ovarian response) or 0.6 mg GnRHa (buserelin), supplemented with 1500 IU hCG on the day of oocyte retrieval (in women with high ovarian response). In cases of excessive ovarian response and/or high risk of OHSS luteal support was not introduced and all good quality blastocysts were frozen. According to significant differences in patients’ age and the number of oocytes in the two groups, matching by age and number of oocytes was performed. No significant differences were observed regarding pregnancy rate per embryo transfer, rate of early pregnancy loss, and livebirth rate per pregnancy between the GnRHa and hCG trigger groups, respectively. A significant difference in the number of developed embryos and blastocysts, as well as the number of frozen blastocysts, was seen in favor of the GnRHa trigger. However, the birth weight in the GnRHa trigger group was significantly lower.

**Conclusion:**

The results of our study support the use of GnRHa for final oocyte maturation in GnRH antagonist IVF cycles in women with high ovarian response. Luteal phase rescue was performed by co-administration of 1500 IU hCG on the day of oocyte retrieval and estradiol and progesterone supplementation. In our experience, such an approach results in a comparable reproductive outcome with hCG trigger group.

## Introduction

Final oocyte maturation is a crucial process that is essential for subsequent fertilization. In the natural cycle, luteinizing hormone (LH) is responsible for the maturation and ovulation of a mature oocyte. As purified LH is not commercially available, recombinant human chorionic gonadotropin (hCG) is used for this purpose. hCG is similar to LH in its chemical structure: they have a common alpha subunit, but distinctly different beta subunits. These differences are responsible for the longer half-life of hCG compared to LH (1).

Ovarian hyperstimulation syndrome (OHSS) is an iatrogenic, potentially lethal complication of ovarian stimulation. An increased number of granulosa cells and luteinization of these cells appear to play a major role in the pathophysiology of OHSS following hCG administration (2). The longer half-life of hCG augments and prolongs luteinizing stimulus, compared to spontaneous LH surge in the natural cycle (3).

The use of a GnRH agonist (GnRHa) instead of recombinant hCG for triggering final oocyte maturation significantly reduces the risk of developing OHSS in GnRH antagonist IVF cycles (4, 5). Because of its short half-life, GnRHa is unlikely to have a long-term stimulatory effect on the early luteal phase and induces quick and complete luteolysis (6).

While triggering oocyte maturation with GnRHa seems to be safe and effective in terms of the risk of developing OHSS and the number of metaphase II oocytes, at the same time, it results in a luteal phase deficiency with lower estradiol and progesterone levels and a shorter luteal phase duration (4, 6, 7). It has long been known that pulsatile secretion of LH in the luteal phase is essential for the undisturbed functioning of the corpus luteum. Premature luteolysis after GnRHa triggering is assumed to be the consequence of excessive negative steroid feedback in the early luteal phase, resulting in suppressed LH release from the pituitary gland (8, 9). As a result, a significantly lower implantation rate and clinical pregnancy rate, and a significantly higher rate of early pregnancy loss were seen in GnRH antagonist cycles after GnRHa triggering, despite luteal phase support with progesterone and estradiol (7, 10, 11). Severe luteal phase deficiency was later confirmed by studies involving donors and recipients after GnRHa and hCG trigger, which observed similar fertilization, implantation, and pregnancy rates (12, 13). Therefore, target groups of patients for GnRHa triggering are oocyte donors, high-responders, and cancer patients undergoing ovarian stimulation for fertility preservation (14).

To date, strategies have been developed in order to rescue defective luteal phase of GnRHa triggered cycles. Some studies have shown that administering 1500 IU hCG 35 hours after ovulation triggering with GnRHa could rescue the luteal phase, resulting in a clinical pregnancy outcome similar to that of ovulation triggered with hCG and without increasing the OHSS rate (15–17). Other studies have also demonstrated the possibility of luteal phase rescue with an aggressive luteal phase support with high doses of progesterone and estradiol (18) or luteal phase supplementation with recombinant LH in conjunction with progesterone (9). Nevertheless, the appropriate luteal phase rescue remains questionable.

Our study aimed to assess the reproductive outcome of GnRHa triggered cycles combined with a modified luteal support (1500 IU hCG at the day of oocyte retrieval) in women with high ovarian response and to compare the outcome with hCG triggered cycles in GnRH antagonist IVF-ICSI procedures.

## Material and Methods

A retrospective cohort database review of the results of GnRH antagonist IVF-ICSI cycles was conducted at the University Medical Centre Ljubljana’s Department of Human Reproduction. The data for the present study were obtained from our institutional database of assisted reproductive technology procedures. For details of data collection and associated ethical considerations, see the Ethics Statement section.

A total of 6126 cycles performed from January 1, 2014, to December 31, 2020, were included in the final analysis. Patients aged 18-43, who underwent a short flexible GnRH antagonist protocol and final oocyte maturation with hCG or GnRHa were included in the present study.

### Ovarian Stimulation and Oocyte Retrieval

The women began injecting recombinant human FSH (Puregon, Organon or Gonal-f, Merck) or highly purified hMG (Menopur, Ferring Pharmaceuticals) on the second day of their menstrual cycle. The dose of gonadotropins was determined by the patient’s age, ovarian reserve, BMI, and previous response to ovarian stimulation (150-300 IU). Once the leading follicle had reached a size of 13 mm in diameter, we initiated cotreatment with the GnRH antagonist cetrorelix 0.25 mg/day (Cetrotide, Merck) and continued up to the day of trigger. Final oocyte maturation was triggered when at least three leading follicles reached 17 mm in diameter. Oocyte maturation was induced depending on the follicle count on the day of trigger. A standard dose of recombinant hCG: 5000, 6500, or 10000 IU (Pregnyl, Organon or Ovitrelle, Merck) was employed in case of fewer than 20 follicles measuring ≥11 mm in diameter. We used 5000 or 10000 IU hCG for final oocyte maturation until 2015. The hCG dose was determined according to the likelihood of developing OHSS: a lower dose of hCG was used in younger and lean patients and in patients with higher number of follicles (more than 10). After 2015, we use 6500 IU hCG in all patients, triggered with hCG. In case of equal or more than 20 follicles measuring ≥11 mm in diameter, we used 0.6 mg single bolus of buserelin (Suprefact, Sanofi).

Transvaginal ultrasound-guided oocyte retrieval was performed 34-36 hours after triggering.

### Luteal Phase Support

In cases of normal or moderate-high ovarian response (fewer than 20 oocytes) and lower risk of developing OHSS, patients with GnRHa trigger received a single subcutaneous bolus of 1500 IU hCG for luteal support one hour after the oocyte retrieval. Due to limited luteinization after GnRHa triggering, we used estradiol and progesterone to support the luteal phase.

The patients administered micronized vaginal progesterone 200 mg (Utrogestan, Medis or Estima, Effik) every 8 hours and oral estradiol 2 mg (Estrofem, Novo Nordisk A/S) every 8 hours from the first day of ovarian puncture until the 12 weeks of gestation.

In cases of excessive ovarian response (more than 20 oocytes) and/or high risk of OHSS, luteal support was not introduced and all good quality embryos were frozen on day 5 or 6.

All patients with hCG trigger received luteal support with micronized vaginal progesterone 200 mg (Utrogestan, Medis or Estima, Effik) every 8 hours from the first day of ovarian puncture until the day of the ultrasound confirmation of a vital pregnancy (i.e., day 28-30 after embryo transfer (ET)).

### Oocyte Fertilization, Embryo Culture, and Embryo Transfer

Fertilization of oocytes was performed using conventional *in vitro* fertilization (IVF) or intracytoplasmic sperm injection (ICSI). Embryos were cultured in G1-Plus and G2-Plus sequential media (Vitrolife) or single-step culture medium SAGE 1-Step (CooperSurgical) until day 3 or 5. A maximum of two embryos were transferred on day 3 or 5 after retrieval, according to national criteria for single embryo transfer. Day 3 cleavage stage embryos were transferred in patients with previous blastocyst development failure and/or with only one or two embryos developed. The blastocysts were graded according to Gardner and Schoolcraft scoring system. Supernumerary blastocysts were frozen on day 5 or day 6.

Biochemical pregnancy was defined by a plasma b-hCG concentration >10 IU/L on day 14 after ET. Clinical pregnancy was defined by ultrasonographic detection of a positive heartbeat on day 30 after ET. The diagnosis of OHSS was made on clinical grounds. The severity of OHSS was graded according to a standardized classification scheme (19).

### Data Collection

Data on the IVF procedures were collected from our internal IVF database, which is updated daily. All 14 Slovenian maternity hospitals systematically collect data on maternal demographic characteristics, medical, gynecological, and reproductive history, prenatal care, pregnancy, delivery, postpartum period, and neonates for each mother-infant pair using the same definitions of variables and the same form of a medical record (National Perinatal Information System, NPIS). The data are sent to the National Institute of Public Health of the Republic of Slovenia by default and were available for this research. The outcomes of the deliveries were obtained from the NPIS. The research was performed according to the Personal Data Protection Act.

### Data Analyses

Data analyses were carried out using SPSS 21.0 (IBM, Armonk, NY, USA). Continuous variables are presented as the mean ± SD and then compared using the Student’s t-test or Mann–Whitney U-test. Values of categorical variables are presented as frequencies and percentages. The chi-square test was used for comparisons between groups. A p value <0.05 was considered significant.

## Results

A total of 6126 IVF-ICSI GnRH antagonist cycles followed by a fresh embryo transfer from January 1, 2014, to December 31, 2020, were included in our study.

There were 829 cycles in the GnRHa trigger group and 5297 cycles in the hCG trigger group. The female cause of infertility was present in 67% of all causes (20% tubal factor infertility, 22% endometriosis, 21% PCOS and hyperprolactinemia, 12% genetic causes or premature ovarian insufficiency, some causes were combined). As many as 33% of patients had undergone uterine surgery (myomas, polyps, uterine anomalies). In 60% there was an abnormal (pathological or suboptimal) semen analysis result. The overall rate of moderate-severe OHSS of early and late onset was 0.9%, without significant differences between the GnRHa and hCG trigger groups after matching the groups according to age and number of oocytes.

**Table 1** presents demographic data of women and reproductive outcomes of GnRHa and HCG triggered cycles.

**Table 1:** Demographic data and IVF-ICSI outcome for women in the GnRHa versus hCG trigger group.

	All N= 6126	GnRHa trigger group N=829	hCG trigger group N=5297	p
**Age years (mean ± SD)**	34,4 ± 4,9	32,1 ± 4,8	34,8± 4,8	**<0,001**
**BMI**	24,2 + 4,9	23,8 ± 4,8	24,3 + 4,9	NS
**Consecutive No. of IVF attempt (mean ± SD)**	1,8 + 1,3	1,8 ± 1,00	1,9 ± 1,3	**<0,001**
**ICSI (%)**	3615 (59%)	512 (65%)	3103 (59%)	**0,012**
**No. of oocytes retrieved (mean ± SD)**	8,8 ± 6,8	15,4 ± 8,1	7,8 ± 6,0	**<0,001**
**No. of immature oocytes (mean ± SD)**	1,5 ± 2,1	2,6 ± 2,1	1,4 ± 1,9	**<0,001**
**No. of degenerated oocytes (mean ± SD)**	0,8 ± 1,3	1,4 ± 1,8	0,7 ± 1,2	**<0,001**
**No. of unfertilized oocytes (mean ± SD)**	1,3 ± 1,8	2,2 ± 2,4	1,1 ± 1,6	**<0,001**
**No. of developed embryos (mean ± SD)**	4,4 ± 4,0	7,8 ± 5,1	3,9 ± 3,5	**<0,001**
**Embryo cleavage rate per no. of normally fertilized oocytes**	98%	98%	98%	NS
**No suitable embryo for ET or freezing**	12%	5%	13%	**<0,001**
**No. of developed blastocysts (mean ± SD)**	1,9 ± 2,6	3,8 ± 2,3	1,7 ± 0,7	**0,001**
**Blastocyst rate**	48%	50%	48%	**0,001**
**No. of frozen blastocysts (mean ± SD)**	1,5 ± 2,5	3,2 ± 2,7	1,2 ± 2,1	**0,005**
**ET performed (%)**	4587 (75%)	551 (66%)	4036 (76%)	**<0,001**
**Single ET (%)**	3024 (65%)	372 (67%)	2652 (65%)	NS
**Pregnancy (% per ET)**	1581 (34%)	230 (41,7%)	1350 (33%)	**0,040**
**Twin birth rate (% deliveries)**	60 (7,5%)*	11 (7,9%)	51 (7,8%)	NS
**Gestation, w (mean ± SD weeks)**	38,4 ± 3,4	38,6 ± 2,9	38,4 ± 3,1	NS
**Pregnancy loss (% per pregnancy)**	334 (21,0%)	48 (20,8%)	286 (21,1%)	NS
**Livebirth (% per pregnancy)**	807 (70%)*	140 (74%)*	669 (69%)*	**0,001**
**Birth weight, g (mean ± SD)**	3142 ± 703	3068 ± 744	3157 ± 693	**0,014**
**Birth weight, 2nd twin, g (mean ± SD)**	2154 ± 635	2085 ± 577	2171 ± 653	**0,026**

SD, standard deviation; BMI, body mass index; ET, embryo transfer; IVF, in vitro fertilization; ICSI, intracytoplasmic sperm injection; p<0,05 – statistical significance; NS, not significant.

*428 pregnancy outcomes were not reported (42 in GnRHa trigger group, 385 in HCG trigger group). p < 0,05 - statistical significance (bold p values).

The women in the GnRHa trigger group were younger and had a lower number of previous IVF attempts. Significant differences were seen regarding the number of all oocytes retrieved, the number of immature, degenerated, and unfertilized oocytes; all numbers were higher in the GnRHa trigger group.

The number of embryos, blastocysts, frozen blastocysts and blastocyst rate were also higher in the GnRHa trigger group. However, the younger age of patients in the GnRHa trigger group could explain the difference in oocyte and blastocyst yield. Analysis revealed that the percentage of cycles without embryos suitable for transfer or freezing was significantly lower in the GnRH trigger group.

ET was performed more often in the hCG trigger group, since GnRHa was used in cases of excessive ovarian response, followed by the “freeze all” protocol. The proportion of single ET and pregnancy loss was similar in both groups. The pregnancy and live birth rates were significantly higher in the GnRHa trigger group.

According to significant differences in patients’ age and the number of oocytes, we decided to design two groups of patients matched by age and number of oocytes. Twenty-nine women from the GnRHa trigger group could not be matched with the hCG trigger group due to a high number of oocytes. The results are shown in the **Table 2**.

**Table 2:** Comparison of IVF-ICSI outcome for women in the GnRHa versus hCG trigger group after matching by age and number of oocytes.

	All N= 1626	GnRHa trigger group N=800	hCG trigger group N=826	p
**Age, years (mean ± SD)**	32,1 ± 4,7	32,0 + 4,6	32,0± 4,8	NS
**Consecutive No. of IVF attempt (mean ± SD)**	1,6 ± 1,0	1,6 ± 1,0	1,6 ± 1,0	NS
**No. of oocytes retrieved (mean ± SD)**	15,3 ± 7,8	15,5 ± 8,1	15,0 ± 8,1	NS
**No. of immature oocytes (mean ± SD)**	2,4 ± 2,8	2,6 ± 2,8	2,6 ± 2,8	NS
**ICSI (%)**	1003 (62%)	512 (64%)	491 (59%)	NS
**No. of degenerated oocytes (mean ± SD)**	1,4 ± 1,8	1,4 ± 1,8	1,4 ± 1,8	NS
**No. of unfertilized oocytes (mean ± SD)**	2,2 ± 2,5	2,3 ± 2,6	2,2 ± 2,5	NS
**No. of developed embryos (mean ± SD)**	7,4 ± 5,1	7,8 ± 5,1	7,1 ± 5,0	**0,002**
**Embryo cleavage rate per no. of normally fertilized oocytes**	98%	98%	98%	NS
**No suitable embryo for ET or freezing**	5%	4%	7%	**0,042**
**No. of developed blastocysts (mean ± SD)**	3,5 ± 3,6	3,9 ± 3,7	3,3 ± 3,4	**0,001**
**Blastocyst rate**	49%	51%	48%	**0,015**
**No. of frozen blastocysts (mean ± SD)**	2,6 ± 3,5	3,2 ± 3,7	1,2 ± 3,4	**0,005**
**ET performed (%)**	1114 (69%)	525 (66%)	589 (71%)	NS
**Single ET (%)**	748 (67%)	394 (67%)	354 (67%)	NS
**OHSS (%)**	26 (1,6%)	14 (1,8%)	12 (1,5%)	NS
**Pregnancy (% per ET)**	450 (30%)	228 (30%)	222 (27%)	NS
**Twin birth rate (% deliveries)**	18 (6%)	11 (7,9%)	7 (5%)	NS
**Gestation, w (mean ± SD weeks)**	38,8 ± 2,3	38,5 ± 2,8	38,8 ± 3,0	NS
**Pregnancy loss (% per pregnancy)**	94 (21%)	48 (20,8%)	46 (20,1%)	NS
**Livebirth (% per pregnancy)**	270 (73%)*	134 (72%)*	137 (75%)*	NS
**Birth weight, g (mean ± SD)**	3145 ± 682	3041 ± 747	3246 ± 600	**0,014**
**Birth weight, 2nd twin, g (mean ± SD)**	2302 ± 600	2085 ± 577	2676 ± 455	**0,026**

SD, standard deviation; BMI, body mass index; ET, embryo transfer; IVF, in vitro fertilization; ICSI, intracytoplasmic sperm injection; OHSS, ovarian hyperstimulation syndrome; p<0,05 – statistical significance. NS, not significant.

*82 pregnancy outcomes were not reported (42 in GnRHa trigger group, 40 in HCG trigger group). p < 0,05 - statistical significance (bold p values).

Most of the differences between the groups disappeared after the matching. However, a significant difference in the number of developed embryos and blastocysts, as well as the number of frozen blastocysts was still seen in favor of the GnRHa trigger. The birth weight of infants in the GnRHa trigger group was found to be significantly lower. No significant differences were seen regarding pregnancy rate per ET, pregnancy loss, and livebirth per pregnancy between the GnRHa and hCG trigger groups.

## Discussion

In our long-term retrospective cohort analysis of GnRH antagonist IVF-ICSI cycles followed by a fresh embryo transfer, we found a statistically significantly higher number of developed embryos, top-quality blastocysts, and frozen blastocyst after GnRHa triggering of final oocyte maturation and modified luteal support with adjuvant low-dose hCG at the time of oocyte retrieval. At the same time, significantly lower birth weight was observed in this group of women. Other reproductive outcomes were comparable between the GnRHa and hCG trigger groups after the matching by age and number of oocytes.

GnRHa triggering reduces OHSS in high-risk patients (14, 18, 20). In controlled ovarian stimulation the most appropriate dosage of FSH in crucial for preventing hyperstimulation. Up until now, the precise tool for predicting the correct starting FSH dosage in high -risk patients has not been found yet. According to Di Paola et al., it has been demonstrated, that clinician’s decision for the appropriate FSH dosage could be supported by the nomogram to adjust the stimulation protocol in order to reduce hyperstimulation risk and reduce the FSH use in high-risk patients. The BMI of the patients is playing the major role in adjusting the dosage next to the hormone levels, age and AFC (21, 22). In selected cases, such as endometriomas, a multidisciplinary approach is warranted (23).

Our results are consistent with several clinical trials using a similar luteal phase rescue protocol after GnRHa triggering. Humaidan et al. in their prospective randomized trial assessed the reproductive outcomes after ovulation induction with GnRHa with a small bolus of hCG (1500 IU), administred on the day of oocyte retrieval. They found that a bolus of hCG in the GnRHa trigger group secured the luteal phase, resulting in comparable reproductive outcomes with the hCG trigger group (16). Similarly, Radesic et al. in their retrospective study of women with a high risk of severe OHSS concluded that GnRHa oocyte maturation in combination with low dose hCG luteal support at the time of oocyte retrieval produces excellent clinical pregnancy rates, while not compromising the ability of GnRHa to prevent severe OHSS (17). Iliodromiti et al.’s international multicenter retrospective case study of 275 women at high risk of OHSS with GnRHa trigger followed by one bolus of 1500 IU hCG 1h after oocyte retrieval, also noted high clinical pregnancy rates and a low incidence of severe OHSS (24).

Our study recorded similar rates of moderate to severe OHSS in both groups of patients after the matching. Nevertheless, the number of follicles measuring <11 mm in diameter on the day of trigger was not frequently recorded, which could be the reason for a similar OHSS rate in both groups. One could speculate that in high responders the use of a GnRHa trigger with co-administration of 1500 IU hCG, instead of classical hCG trigger, enables subsequent fresh ET with the similar reproductive outcome and higher frozen blastocyst rate, as was already concluded by Iliodromiti et al. (24).

The association between ovarian stimulation, followed by fresh ET and adverse obstetric outcomes, such as lower birth weight and higher risk of preterm delivery has long been known (25–28). However, it is not yet fully understood whether this risk differs among different stimulation protocols. Imudia et al. in their retrospective cohort study assessed the impact of elevated serum estradiol levels on the day of hCG administration during controlled ovarian hyperstimulation for IVF on the likelihood for small for gestational age (SGA), preeclampsia, and preterm delivery in singleton pregnancies. The odds of delivering SGA infants and developing preeclampsia was nine- and five-fold greater, respectively, in patients with elevated peak serum estradiol levels as compared with patients with lower serum estradiol levels (29). Our study established an increased risk of lower birth weight of infants in the group of women triggered with GnRHa. There are no data on the effect of a single dose of GnRHa on the birth weight of children. Lower birth weight is probably the consequence of higher serum estradiol concentrations in the GnRHa trigger group. Since there was higher number of follicles and oocytes in this group, the assumption of higher estradiol serum levels seemed reasonable and consistent with the literature.

Recently, some promising studies on hCG-based only luteal phase support in IVF patients have been published. These studies investigated the effect of luteal phase support with one bolus of 1500 IU hCG 2 days after oocyte retrieval (30) or with 1500/1000 IU hCG on the day of oocyte retrieval plus 1000/500 IU hCG 4 days later, depending on the follicle count on the day of trigger (31). Both studies found sufficient endogenous progesterone production, without the need for progesterone and estradiol replacement. Luteal phase support without vaginal progesterone supplementation appears to be more patient-friendly. However, controlled studies with a large number of patients are needed prior to the widespread use in clinical practice.

The results of our study support the use of a GnRHa trigger protocol with luteal phase rescue with co-administration of one bolus of 1500 IU hCG on the day of oocyte retrieval for women with high ovarian response.

Although the cumulative pregnancy rate was not assessed in our study, we may presume that it is higher in the GnRHa trigger group, as a result of significantly more frozen blastocysts obtained.

The treatment protocol for high responders is well established and accepted at our department. In cases of extreme ovarian response, GnRHa trigger is followed by a ‘freeze all’ policy to avoid any risk of developing OHSS (32).

The limitations of our study are its retrospective nature, lack of data on the total follicle count, number of MII oocytes, and mild OHSS rate. Basic characteristics such as age, number of consecutive IVF attempts, ICSI rate and so on differ between the two groups.

## Data Availability Statement

The raw data supporting the conclusions of this article will be made available by the authors, without undue reservation.

## Ethics Statement

Ethical approval was not provided for this study on human participants because the data for the present study were obtained from our own institutional database of assisted reproductive technology procedures. Collection and analysis of these data in an anonymized form is allowed by the Personal Data Protection Act (Article 17, Official Gazette of the Republic of Slovenia No 94/07, 2004) and by the Healthcare Databases Act (Official Gazette of the Republic of Slovenia No 65/00, 2000; No 47/15, 2015; 31/18, 2018). The collection of anonymized data for observational studies in standardized treatments and the usual management of patients is allowed by the National Medical Ethics Committee of Slovenia (0120-174/2018/6). Before inclusion to IVF procedures, each patient signs an informed consent for the procedure and anonymized data collection and analysis for research purposes. The present research was performed in accordance with relevant guidelines and regulations. The patients/participants provided their written informed consent to participate in this study.

## Author Contributions

Conceptualization, DA, SK, and EVB. Methodology, DA, EVB, MS, and SK. Validation, DA and SK. Formal analysis, PC, MS, and SK. Investigation, all authors. Writing – original draft preparation, DA. Writing – review and editing, MS, EVB, and SK. Visualization, DA. Supervision, EVB and SK. All authors have read and agreed to the final version of the manuscript. All authors are accountable for all aspects of the work in ensuring that questions related to the accuracy or integrity of any part of the work are appropriately investigated and resolved.

## Funding

This research received no specific grant from any funding agency in the public, commercial, or non-profit sectors.

## Conflict of Interest

The authors declare that the research was conducted in the absence of any commercial or financial relationships that could be construed as a potential conflict of interest.

## Publisher’s Note

All claims expressed in this article are solely those of the authors and do not necessarily represent those of their affiliated organizations, or those of the publisher, the editors and the reviewers. Any product that may be evaluated in this article, or claim that may be made by its manufacturer, is not guaranteed or endorsed by the publisher.
